# 701 Effect of Methamphetamine Use on Transthoracic Echocardiographic Findings in Burn Patients

**DOI:** 10.1093/jbcr/irae036.246

**Published:** 2024-04-17

**Authors:** John H Sojka, Elias Jbeily, Taylor Silva, Samveda Rukmangadhan, Jason Heard, Soman Sen, Tina L Palmieri, Kathleen S Romanowski

**Affiliations:** Arizona Burn Center, Phoenix, AZ; University of California, Davis, Antelope, CA; UC Davis Medical Center, Cameron Park, CA; University of California, Davis, Petaluma, CA; University of California - Davis Hosp, Sacramento, CA; UC Davis, Sacramento, CA; UC Davis, Shriners Children's Northern California, Sacramento, CA; Arizona Burn Center, Phoenix, AZ; University of California, Davis, Antelope, CA; UC Davis Medical Center, Cameron Park, CA; University of California, Davis, Petaluma, CA; University of California - Davis Hosp, Sacramento, CA; UC Davis, Sacramento, CA; UC Davis, Shriners Children's Northern California, Sacramento, CA; Arizona Burn Center, Phoenix, AZ; University of California, Davis, Antelope, CA; UC Davis Medical Center, Cameron Park, CA; University of California, Davis, Petaluma, CA; University of California - Davis Hosp, Sacramento, CA; UC Davis, Sacramento, CA; UC Davis, Shriners Children's Northern California, Sacramento, CA; Arizona Burn Center, Phoenix, AZ; University of California, Davis, Antelope, CA; UC Davis Medical Center, Cameron Park, CA; University of California, Davis, Petaluma, CA; University of California - Davis Hosp, Sacramento, CA; UC Davis, Sacramento, CA; UC Davis, Shriners Children's Northern California, Sacramento, CA; Arizona Burn Center, Phoenix, AZ; University of California, Davis, Antelope, CA; UC Davis Medical Center, Cameron Park, CA; University of California, Davis, Petaluma, CA; University of California - Davis Hosp, Sacramento, CA; UC Davis, Sacramento, CA; UC Davis, Shriners Children's Northern California, Sacramento, CA; Arizona Burn Center, Phoenix, AZ; University of California, Davis, Antelope, CA; UC Davis Medical Center, Cameron Park, CA; University of California, Davis, Petaluma, CA; University of California - Davis Hosp, Sacramento, CA; UC Davis, Sacramento, CA; UC Davis, Shriners Children's Northern California, Sacramento, CA; Arizona Burn Center, Phoenix, AZ; University of California, Davis, Antelope, CA; UC Davis Medical Center, Cameron Park, CA; University of California, Davis, Petaluma, CA; University of California - Davis Hosp, Sacramento, CA; UC Davis, Sacramento, CA; UC Davis, Shriners Children's Northern California, Sacramento, CA; Arizona Burn Center, Phoenix, AZ; University of California, Davis, Antelope, CA; UC Davis Medical Center, Cameron Park, CA; University of California, Davis, Petaluma, CA; University of California - Davis Hosp, Sacramento, CA; UC Davis, Sacramento, CA; UC Davis, Shriners Children's Northern California, Sacramento, CA

## Abstract

**Introduction:**

Methamphetamine use adversely effects the cardiovascular system leading to pulmonary hypertension and heart failure (HF) due to non-ischemic dilated cardiomyopathy with reduced ejection fraction (EF). Despite this, many methamphetamine (MA) users with HF have preserved EF and the adverse effects of their MA use may be overlooked. Subclinical cardiac dysfunction associated with MA use may become clinically apparent in patients experiencing a significant physiological stress like burn injury. We hypothesize that transthoracic echocardiogram (TTE) findings and outcomes of MA positive patients will be worse than those of MA negative patients.

**Methods:**

Following IRB approval, data was collected on all patients admitted for burn injuries who had admission TTEs between January 2015 and December 2019. Patients were divided into two groups based on admission drug screen positivity for MA. Data collected included TTE findings, patient demographics, percent total body surface area (%TBSA) burned, length of stay (LOS), hospital charges, and mortality. The two groups were then matched based on age and burn %TBSA. Analysis on the total population and the matched data set was conducted with SAS statistical software using Chi-square, Fisher Exact, and Wilcoxon 2-sample tests.

**Results:**

A total of 417 patients with TTEs were included, 295 MA negative and 122 MA positive (29.3%). In the total population, MA positive patients were younger (p< 0.0001), had larger burns (p=0.003), more inhalation injury (p=0.002), less aortic regurgitation (p< 0.0001), higher left ventricular EF (p=0.01), higher hospital charges (p=0.003), and lower mortality (p=0.02) than their MA negative counterparts. In the group matched for age and %TBSA, there was no significant difference in inhalation injury, sex, race, or BMI between groups. The MA negative group had significantly more patients with aortic regurgitation (p=0.02). All other TTE findings were not significantly different. No significant differences were found in LOS (p=0.25), operations (p=0.08), charges (p=0.19), or mortality (p=0.14). The circumstances of burn injuries were significantly different (p=0.03), where MA positive patients were more likely to be burned as a result of suspected assault or self-injury and MA negative patients were more likely to have accidental workplace related burns.

**Conclusions:**

Patients who tested positive for MA on admission were younger and had larger burns. When controlled by matching, there were no differences in TTE findings or clinical outcomes related to testing MA positive.

**Applicability of Research to Practice:**

Admission drug screen positivity for methamphetamine alone may not be an indication for obtaining TTE.

